# Evaluation of Plant Growth Promoting Bacteria Strains on Growth, Yield and Quality of Industrial Tomato

**DOI:** 10.3390/microorganisms9102099

**Published:** 2021-10-05

**Authors:** Nikolaos Katsenios, Varvara Andreou, Panagiotis Sparangis, Nikola Djordjevic, Marianna Giannoglou, Sofia Chanioti, Panagiota Stergiou, Maria-Zacharoula Xanthou, Ioanna Kakabouki, Dimitrios Vlachakis, Snezana Djordjevic, George Katsaros, Aspasia Efthimiadou

**Affiliations:** 1 Department of Soil Science of Athens, Institute of Soil and Water Resources, Hellenic Agricultural Organization—Demeter, 14123 Lycovrissi, Greece; nkatsenios@gmail.com (N.K.); pansparangis@gmail.com (P.S.); 2 Institute of Technology of Agricultural Products, Hellenic Agricultural Organization—Demeter, 14123 Lycovrissi, Attica, Greece; vandreou@chemeng.ntua.gr (V.A.); giannoglou@chemeng.ntua.gr (M.G.); schanioti@gmail.com (S.C.); ster.panagiota@gmail.com (P.S.); mxanthou@itap.com.gr (M.-Z.X.); gkats@chemeng.ntua.gr (G.K.); 3 Agrounik d.o.o., Milana Uzelca 11, 11080 Belgrade, Serbia; nikola.djordjevic@agrounik.rs; 4 Laboratory of Agronomy, Department of Crop Science, Agricultural University of Athens, 11855 Athens, Greece; i.kakabouki@gmail.com; 5 Laboratory of Genetics, Department of Biotechnology, School of Applied Biology and Biotechnology, Agricultural University of Athens, 11855 Athens, Greece; dimvl@aua.gr; 6 Laboratory of Molecular Endocrinology, Center of Clinical, Experimental Surgery and Translational Research, Biomedical Research Foundation of the Academy of Athens, 11527 Athens, Greece; 7 Department of Informatics, Faculty of Natural and Mathematical Sciences, King’s College London, Strand, London WC2R 2LS, UK; 8 Biounik d.o.o., Milana Uzelca 11, 11080 Belgrade, Serbia; biounik1@gmail.com

**Keywords:** plant growth promoting bacteria, strains, biostimulants, industrial tomato, brix, antioxidant activity

## Abstract

Plant growth promoting bacteria (PGPB) are used as biostimulants to improve the growth and yield as well as the quality of crops. In the present study, nine strains of PGPB and one solid mix consisting of two of them were evaluated on the cultivation of industrial tomato under specific soil and climatic conditions. The results showed that *Bacillus licheniformis* treatment increased dry weight of the tomato plants by 39%, and the photosynthetic rate was increased by *Priestia megaterium* 9.9%. The application of *Bacillus subtilis*, *Bacillus amyloliquefaciens*, *Priestia megaterium*, and *Bacillus licheniformis* increased mean fruit weight per plant 26.78–30.70% compared to that of control. Yield per plant was increased 51.94% with the use of *Bacillus licheniformis* compared to that of control. The quality of the fruits in nearly every bacteria strain was improved. *Bacillus pumilus* and the mix of *Priestia megaterium* and *Azotobacter chroococcum* (1:1) increased the most total soluble solids in the tomato fruits (4.70° Brix), and *Priestia megaterium* increased content in lycopene and total carotenoids by 52.8% and 25%, respectively; *Bacillus pseudomycoides* increased Pectin methylesterase (PME) activity (24.94 units/mL), and *Bacillus* *mojavensis*, along with the mix of *Priestia megaterium* and *Azotobacter chroococcum*, increased Poligalacturonase (PG) activity the most (30.09 and 32.53 units/mL, respectively). Most of the bacteria strains presented an increased antioxidant activity significantly better that that of the control up to 31.25%. The results of this study confirmed that the use of PGPB as biostimulants can improve the yield and the quality of industrial tomato.

## 1. Introduction

More and more sustainable cultivation practices are introduced and implemented to modern agriculture. Current research focuses to investigate and enhance these practices to achieve sustainable and productive results. The implementation of plant biostimulants in many plant species is a promising practice that was used over the last decades [1,2]. Plant biostimulants can be microorganisms such as species and strains of beneficial bacteria as well as any substances that can stimulate the performance of plants by increasing their nutrition efficiency, abiotic stress tolerance, and their quality traits [3,4,5]. Plant growth promoting bacteria (PGPB) is a promising category of biostimulants that showed positive interaction in some plants, and thus, gained the interest of the researchers worldwide. PGPB can usually be found in the soil environment and can move to rhizosphere and rhizoplane as well as to some aerial parts of the plants [6].

Methods of application can be the inoculation of bacteria either on the seeds or roots of seedling, direct soil, and foliar application [4,7,8,9]. Some of their functions that were reported are the ability to produce growth regulators [10,11], the creation of antagonistic environment for phytopathogens [12], the ability to change or release hormones as well as produce volatile organic compounds that stimulate plant growth, and increase the availability and uptake of nutrients by the plants and the abiotic stress tolerance [7,13]. Moreover, studies showed that there are strains of PGPB responsible for N_2_ fixation [8,14], and others that can cause the solubilization of mineral phosphates [15].

Efthimiadou et al. [3] evaluated several PGPB biostimulants in terms of growth, yield, and quality characteristics of maize plants as well as the application method that was used. They concluded that the soil inoculation of bacteria presented better results than the control. Rojas–Tapias et al. [9] found that the shoot length and dry weight at the first level of salinity were increased when maize roots were inoculated with strains of *Azotobacter*.

After a large number of studies that demonstrated the positive effect of PGPB on plant growth were conducted, researchers focused on further investigation of specified strains of the bacteria to evaluate them and see whether they could be applied in common agricultural practices. For instance, strains AC1 and AC10 of *Azotobacter chroococcum* were reported to promote seed germination and cotton growth [14], while strains M14, M15, M22, M26, and M37 of the same species were found to contribute to the phosphate solubilizing in an experiment of wheat [15]. The strains *Pseudomonas putida* BA-8 and Bacillus subtilis OSU-142 were used to increase yield and nutrition of apple trees [16]. *Herbaspirillum seropedicae* strain Z67 was found to combined with humic substances and can increase maize grain production by 65% [17]. *Azotobacter chroococcum* Strain 5 and *Azospirillum lipoferum* Strain 21 increased growth and yield of canola [8]. Moreover, *Pseudomonas fluorescens* strain Pf4 and *P. aeruginosa* strain Pag increased the antifungal compounds in peas [12].

Research should also focus on the potential effect PGPB could have on the quality of the final products. Efthimiadou et al. [3] concluded that *B. subtilis* treatment increased total solids content in harvested maize seeds by 92%, as well as crude fiber content by 46% compared to that of control. The results confirmed that the use of PGPB could contribute as a new cultivation practice for sustainable growth, productivity, and quality of grain crops. PGPB had a positive effect on total soluble solids (TSSs) of tomato plants according to a pot experiment by Dudás et al., [18]. Also, it was proven that PGPB can also affect quality characteristics, such as lycopene, sweetness index, β-carotene, and lutein [19,20,21].

The aim of this study was to investigate the effect of nine plant growth promoting bacteria and one solid mix consisted of two of them, on the cultivation of industrial tomato under specific soil and climatic conditions. Measurements of plant growth, physiology of the plant, yield, and quality characteristics of industrial tomato were conducted to evaluate the different strains of the PGPB used.

## 2. Materials and Methods

### 2.1. Experimental Site and Design

**Figure 1:**
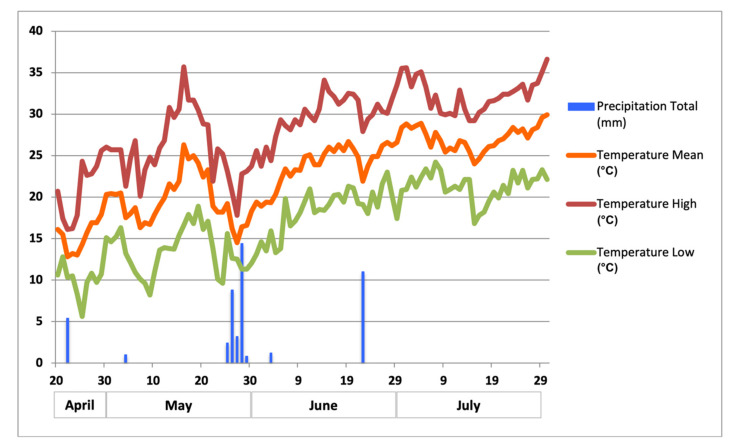
Daily mean, high and low temperature, and precipitation at Oropos during experimental period.

A field experiment was established at Oropos (38°18′ N, 23°45′ E, Altitude 45 m), in the Prefecture of Attica, Greece. Tomato certified seeds of *Solanum lycopersicum* L. cv. Rio Grande (HortuSì Srl, Italy, imported by Gemma S.A., Athens, Greece) were used to create the young tomato plants. Tomato plants were transplanted on 20 April 2020, and they harvested on 30 July 2020, 101 days after transplanting (DAT). Daily mean, maximum, and minimum temperature and precipitation during the experimental period are presented at Figure 1.

The experiment followed a completely randomized design, with 11 treatments (9 strains of PGPB, 1 Mix and control). The PGPB that were used were 7 species of *Bacillus*, 1 species of *Priestia* and 1 species of *Azotobacter.* The sequence data of the seven *Bacillus* species and the *Priestia* were submitted to GenBank of NCBI database (Table 1. The Mix consisted of *Priestia megaterium* B004 (3.4 × 10^7^ CFU/mL) + *Azotobacter chroococcum* A004 (1.3 × 10^7^ CFU/mL) at a ratio of mixing 1:2 with neutral pH (6.8–7.2). The carrier of this mix was zeolite and the final form of the mix was solid. The experiment had three replications and every experimental plot consisted of an area of 6m^2^. The space between rows was 75 cm and between plants in the row 50 cm.

The solution of the PGPB was diluted with tap water (1:100), and then it was added to the soil close to the tomato plants. The application rate of PGPB was 7 lt/ha. The application was conducted on 16 May, 36 DAT.

The weather conditions (daily mean, high and low temperature, and precipitation) during the experimental period were retrieved from the NOANN network of the National Observatory of Athens [22].

A soil sample was collected from four representative cores of the experimental field in depth of 0–30 cm, three weeks before transplanting. The elements Ca^2+^, Mg^2+^, and K^+^ were determined by atomic absorption spectrometry [23], Zn^2+^, Mn^2+^, Cu^+^, and Fe^3+^ were determined by atomic absorption spectrometry using DTPA [24]. Available B was determined using a spectrophotometer, using azomethine-H as the color (yellow) development reagent [25]. Total nitrogen was determined with ISO, 1995 (11261) [26], organic carbon according to ISO, 1998 (14235) [27], available phosphorus with ISO, 1994 (11263) [28], soil texture was determined using the method of Bouyoucos [29], the moisture content was determined in a furnace at 105 °C for 24 h, and the value of pH was measured with a pH-meter equipped with glass electrode in the saturated paste extract. Total salts were calculated using the results of electrical conductivity and the saturation percentage of the soil samples. Electrical conductivity was determined in an aqueous extract of soil according to ISO 11265:1994 [30]. The results of soil physical and chemical properties are presented in Table 2.

### 2.2. Cultivation of Bacteria

The bacterial strains are part of the collection of Agrounik d.o.o. (Belgrade-Zemun, Serbia). The bacteria were identified by sequencing 16 rDNA. For the isolation of genomic DNA, “Quick-DNA Fungal/Bacterial Miniprep Kit” kit (Zimo Research) was used. Isolated genomic DNA was further used for gene amplification by polymerase chain reaction (PCR). PCR amplification was performed in 25 µL of the reaction mixture containing 1 µL of DNA, 12.5 µL OneTaq® 2X Master Mix with Standard Buffer (New England biolabs, Ipswich, MA, USA), 0.5 µM of each primer and nuclease-free PCR water supplemented to 25 µL. The universal primers UN116SF and UN116SR were used. On the ‘T100 Thermal cycler’ Biorad™, PCR reactions were performed according to the following protocol: initial denaturation at 95 °C for 3 min, 40 cycles consisting of denaturation at 95 °C for 15 s, primer hybridization at 53 °C for the 30 s, and 72 °C extensions for 90 s, and final extension at 72 °C for 90 s. The length of the PCR products was determined by horizontal electrophoresis (20 min at 100V) at 2% agarose. PCR products were purified via the ‘DNA Clean & Concentrator ™’ kit (Zymo Research) and sent for sequencing to the “Macrogen” sequencing service (Netherlands). PCR product sequences were analyzed using the BLAST nucleotide sequence search program on the National Center for Biotechnology Information (NCBI) website. The sequences were submitted to the NCBI database and GenBank accession numbers were received.

Different colonies were seeded in 100 mL of TSB and *Azotobacter* medium for 24 h, with optical density between 0.3–0.35. After this process, 2% of the inoculum was seeded in 3L of the medium. *Bacillus* and *Priestia* species were cultivated in Tryptic Soy Broth (TSB) and grown under aerobic condition at 32 °C with shaking at 200 rpm [31]. *Azotobacter chroococcum* was cultivated in *Azotobacter* medium (Azotobacter Agar—Mannitol, HiMedia Laboratories Pvt. Ltd., Maharashtra, India) and grown at 30 °C with shaking at 180 rpm for 72 h. At the end of fermentation, bacterial strains were tested for their optimal growth (Colony-forming unit—CFU), pH, and production of plant hormone auxin by colorimetric analysis [32]. Specifically for the Strain B002 of the *Bacillus amyloliquefaciens*, in addition to identification via the 16s gene, we also identified them via two housekeeping genes (EFtu and RecA) to validate the result that is *Bacillus amyloliquefaciens* and not *B. velezensis*.

### 2.3. Measurements

Dry weight and physiology measurements were conducted 66, 80, and 94 DAT. Dry weight was measured with a precision balance after the samples (whole plants including tomatoes) were oven-dried at 70 °C for three days to measure the weight in grams per plant. Physiology measurements of photosynthetic rate (μmol CO_2_ m^−2^ s^−1^) and transpiration rate (mmol H_2_O m^−2^ s^−1^) were conducted with an LCi Leaf Chamber Analysis System (ADC, Bioscientific, Hoddesdon, UK) during midday hours on fully expanded leaves when the sky was clear. Collection of plant material complied with relevant institutional, national, and international guidelines and legislation.

### 2.4. Evaluation of Quality Parameters of Tomatoes 

#### 2.4.1. Physicochemical Parameters 

Major physicochemical parameters of fresh tomatoes were determined for all studied samples based on methods described by Andreou et al. [33]. Total soluble solids (°Brix- KERN Digital Refractometer, KERN & SOHN GmbH, Balingen, Germany), pH (ORION 188 ion analyzer model EA 940, ORION-scientific, Limena, Padova, Italy), and color (CIELab scale-Minolta CR-300 colorimeter) of obtained tomato juices were evaluated. The moisture content of fresh tomatoes was measured by drying at 110 °C for 24 h (Memmert, B50 type, Memmert GmbH + Co. KG) and the ash was determined by drying (Nabertherm GmbH, Lilienthal, Germany) at 550–600 °C for 5 h.

#### 2.4.2. Determination of Endogenous Enzymes Activity of Tomatoes

Endogenous enzymes activities, such as pectinmethylesterase (PME) and polygalacturonase (PG), were determined following the method described by Andreou et al. [33]. PME was measured using a titrimetric method and PG activity spectrophotometrically (Photometer XD 7500, Lovibond, Germany) based on the detection of polygalacturonic acid hydrolysis with cyanoacetamide at 276 nm. Both enzymes’ activities were estimated and expressed as unit/mL.

#### 2.4.3. Determination of Intracellular Bioactive Compounds of Tomatoes

The major intracellular bioactive compounds of tomatoes that were determined were the concentration of total carotenoids (mg/g dw), lycopene content (mg/g dw), total phenolic compounds (mg caffeic acid/g dw), and the antioxidant capacity (mg Trolox/g dw). For all studied samples, tomatoes were homogenized, deep frozen (−80 °C), dried (−52 °C, 0.080 mbar for 48 h using a Thermo Savant MODULYOD-230 freeze dryer (Thermo Fisher Scientific, San Jose, CA, USA), and then stored until further analysis.

#### 2.4.4. Extraction and Quantification of Total Carotenoids

Extraction of total carotenoids from dried tomato samples was performed by using as solvent a mixture solution hexane:acetone:ethanol (50:25:25) following the procedure described by Andreou et al. [34]. Two (2) grams of dried tomato samples were mixed and stirred with 20 mL of organic mixture for 30 min at room temperature. Cold, doubly distilled water (10 mL) was added, and the suspension was agitated for an additional 5 min. The solution was allowed to stand for 15 min for separation of polar and nonpolar layers. The content of total carotenoids (TC) was determined spectrophotometrically at 470 nm (A_470_), 663 nm (A_663_), and 647 nm [35] and was estimated using the Lichtenthaler equations:

(1) $$Chlorophyll\;a=12.25\times A_{663\;\mathrm{nm}}-2.79\times A_{647\;\mathrm{nm}}$$

(2) $$Chlorophyll\;b=21.50\times A_{647\;\mathrm{nm}}-5.10\times A_{663\;\mathrm{nm}}$$

(3) $$\begin{array}{c} Total\;carotenoids=(1000\times A_{470\;\mathrm{nm}}-1.82\times Chlorophyll\;a-\\ 85.02\times Chlorophyll\;b)/228 \end{array}$$

#### 2.4.5. Lycopene Extraction and Quantification by HPLC

Nonpolar tomato extracts were further analyzed by high performance liquid chromatography (HPLC) to identify individual carotenoids, such as lycopene, according to Dermesonlouoglou et al., [36]. The HPLC equipment fitted with an Agilent Zorbax Eclipse Plus C18 (250 mm × 4.6 mm) of particle size 5 μm. The mobile phase was isocratic consisted of methanol:tetrahydrofuran:water at ratios 67:27:6 with flow rate 2 mL/min. Detection was performed at 472 nm. The method was calibrated by an external standard. Lycopene content was expressed as mg/g dw.

#### 2.4.6. Extraction and Quantification of Total Phenolic Compounds

Extraction of total phenolic compounds (TPC) from dried tomato samples was carried out as described by Andreou, et al. [34], with some modifications. TPC were extracted with suitable liquid-to-solid ratio (10:1) allowing to maintain a homogenous solid-liquid extraction. Ten grams of dried tomato product were mixed with 60% ethanol solution and were agitated for 30 min. The extraction temperature was controlled (20 ± 2 °C). After the extraction, the samples were centrifuged and then the supernatant was stored at −20 °C. Total phenolic compounds were estimated following the Folin–Ciocalteu method. Tomato extracts of 100 μL were mixed with 7.9 mL of distilled water and 500 μL of Folin–Ciocalteu solution. Over the next 1.5 min, 1.5 mL of saturated sodium carbonate solution (Na_2_CO_3_) was added and then the solution was heated in a water bath at 40 °C for 30 min. Absorbance of the solution was measured at 765 nm. Total phenol concentration was expressed as mg of caffeic acid equivalence/g dw (CAE/g dw). Samples were analyzed in triplicate.

#### 2.4.7. Antioxidant Capacity

Antioxidant capacity in dried tomato samples was determined spectrophotometrically as described by Chanioti and Tzia, [37]. An amount of 3.9 mL 2,2-diphenyl-1picrylhydrazyl (DPPH) solution (0.0025 g per 100 mL of methanol) was mixed with 100 μL of 60% ethanol extract of tomato products (extraction as described in Section 2.4.6) and incubated at 25 °C for 30 min in darkness, and the absorbance at 515 nm was measured after 30 min. Trolox (6-hydroxy-2,5,7,8-tetramethylchroman-2-carboxylic acid) was used as a standard. The antioxidant capacity was reported as mg Trolox/g dw. Samples were analyzed in triplicate.

### 2.5. Statistical Analysis

One-way analysis of variance (ANOVA) was used to evaluate the effect of PGPB application. The experimental data were analyzed using IBM SPSS software ver. 24 (IBM Corp., Armonk, NY, USA). PCA is a dimensionality-reduction method that is often used to reduce the dimensionality of large data sets by transforming these huge set of variables into a smaller one that still contains the accurate information. By computing the eigenvector allow to find the principal components that are in significance. In this point of view, in this study, it was necessary to normalize data before performing PCA. Nine parameters were set (Yield, MW, PME, PG, Brix, Yield, TPC, TC, Lycopene), making the analysis of their data much easier when correlating samples with variables. The comparisons of means were calculated using Duncan test at the 5% level of significance (*p* ≤ 0.05). Multivariate analysis was conducted by means of principal component analysis (PCA) by using STATISTICA 7 (Statsoft Inc., Tulsa, OK, USA).

## 3. Results

The effect of nine strains of plant growth promoting bacteria (PGPB) and a mix of two of them on the dry weight, the photosynthetic rate, and the transpiration rate measurements, as well as on the mean fruit weight and the yield per plant of the industrial tomato was studied.

### 3.1. Plant Growth

At the first measurement (66 DAT) of dry weight, the treatments of *B. pseudomycoides, Mix* and *B. licheniformis* (336 g, 334 g, and 328 g, respectively) resulted in the highest values with statistically significant differences compared to all other treatments (Table 3). Moreover, all PGPB treatments except *B. velezensis, A. chroococcum*, and *P. megaterium* had statistically significant differences compared to that of *control*. At the second measurement (80 DAT), the highest values were achieved in *B. licheniformis* (442 g) and *Mix* (436 g) treatments, with no statistically significant differences among them, whereas many PGPB treatments had no statistically significant differences compared to control. At the final measurement (94 DAT), *B. licheniformis* (761 g) led to the highest value of dry weight per plant, followed by *B. subtilis* (722 g), with statistically significant differences compared to control. All PGPB that were used, except *B. mojavensis B. mojavensis* and *B. velezensis*, increased dry weight.

### 3.2. Physiology Measurements

Concerning the photosynthetic rate measurement (Table 4), the treatments of *P. megaterium* (18.03 μmol CO_2_ m^−2^ s^−1^) and *B. subtilis* (17.96 μmol CO_2_ m^−2^ s^−1^) resulted in the highest values with statistically significant differences compared to that of the other treatments, excluding *B. licheniformis, B. pseudomycoides*, and *Mix* (Table 4). *P. megaterium* (18.48 μmol CO_2_ m^−2^ s^−1^) was the treatment with the highest photosynthetic rate and for the second measurement (80 DAT), followed by *B. amyloliquefaciens* (18.00 μmol CO_2_ m^−2^ s^−1^). All PGPB treatments, except *B. pumilus*, were significantly different compared to that of the untreated plants. At the third measurement (94 DAT), *P. megaterium* was still the treatment with the highest value (18.27 μmol CO_2_ m^−2^ s^−1^), followed by *B. velezensis* and *A. chroococcum* (18.02 and 17.82 μmol CO_2_ m^−2^ s^−1^, respectively), which reached values that were statistically significantly higher compared to that of control.

Transpiration rate of plants (Table 5) treated with *B. subtilis* (3.71 mmol H_2_O m^−2^ s^−1^) was statistically significantly higher than the other treatments, except *P. megaterium* (3.55 mmol H_2_O m^−2^ s^−1^) *Mix* (3.40 mmol H_2_O m^−2^ s^−1^) and *B. amyloliquefaciens* (3.38 mmol H_2_O m^−2^ s^−1^), at the first measurement (66 DAT). At the second measurement (80 DAT), *B. velezensis* (3.25 mmol H_2_O m^−2^ s^−1^ g) achieved the highest value, followed by *B. pumilus* (3.23 mmol H_2_O m^−2^ s^−1^), with statistically significant differences compared to *control*. All PGPB that were used, except *B. amyloliquefaciens, A. chroococcum*, and *P. megaterium*, had statistically significant differences compared to that of *control*.

### 3.3. Yield

**Figure 2:**
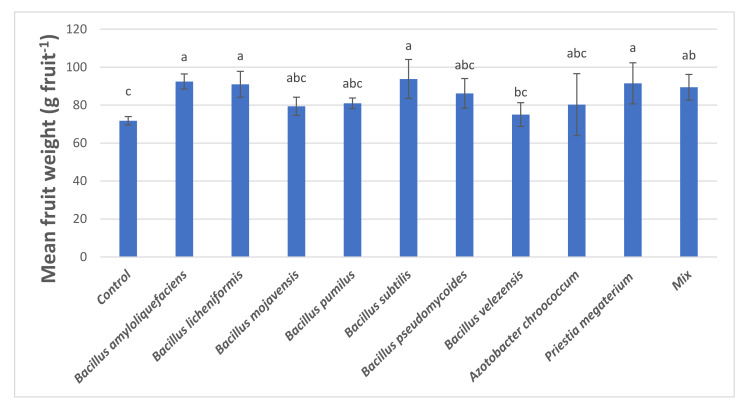
Mean fruit weight of harvested tomatoes. Mix: Mix of *Priestia megaterium* B004 + *Azotobacter chroococcum* A004 with zeolite as a carrier. Means followed by same letter for treatments are not significantly different according to Duncan test (*p* < 0.05). Values presented are mean values of three replicates ± standard deviation. F value of ANOVA: 2.585 (Significance level: *p* < 0.001).

The use of PGPB increased the mean fruit weight of tomatoes at the harvest (Figure 2). The treatment of *B. subtilis* (93.77 g fruit^−1^) gave the highest weight, followed by *B. amyloliquefaciens* (92.41 g fruit^−1^), *Priestia megaterium* (91.49 g fruit^−1^), and *B. licheniformis* (90.95 g fruit^−1^) with statistically significant differences compared to *control*. Moreover, the treatment of *Mix* (89.40 g fruit^−1^) was statistically significantly different compared to that of *control*, while the rest of the PGPB treatments, although all of them presented higher values of the *control*, their differences were statistically nonsignificant.

**Figure 3:**
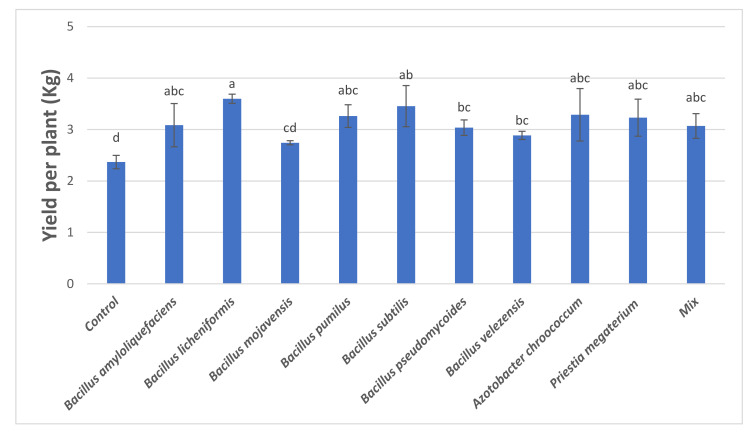
Mix: Mix of *Priestia megaterium* + *Azotobacter chroococcum*. Means followed by same letter for treatments are not significantly different according to Duncan test (*p* < 0.05). Values presented are mean values of three replicates ± standard deviation. F value of ANOVA: 4.367 (Significance level: *p* < 0.001).

The soil application of PGPB on tomato processing plants resulted in yield increase per plant for all studied treatments, except *B. mojavensis*, with statistically significant differences compared to that of *control* (Figure 3). The treatment of *B. licheniformis* (3.60 kg) led to the highest yield, followed by *B. subtilis* (3.45 kg). The treatments of *Bacillus amyloliquefaciens* (3.09 kg)*, Bacillus pumilus* (3.26 kg)*, Bacillus pseudomycoides* (3.04 kg)*, Bacillus velezensis* (2.89 kg)*, Azotobacter chroococcum* (3.29 kg)*, Priestia megaterium* (3.23 kg), and *Mix* (3.07 kg), had higher yield compared to that of control (2.37 kg), with statistically significant differences, but the differences were nonsignificant among them.

### 3.4. Quality Characteristics of the Harvested Tomatoes

The effect of PGPB on several quality characteristic of harvested tomatoes is presented in Table 6. Moisture, pH value, and ash were not significantly (*p* > 0.05) affected from the soil application of PGPB and ranged between 94.5–95.3 ± 0.4%, 4.42–4.51 ± 0.4 and 0.46–0.57%, respectively, for all studied samples.

All PGPB treatments resulted in higher total soluble solids (°Brix) with statistically significant differences from control samples (4.0 ± 0.1) by at least 10% (all values were higher for PGPB samples by 10–19% compared to that of control). *B. licheniformis, B. pumilus, Mix, P. megaterium*, *B. amyloliquefaciens*, and *B. mojavensis* treatments led to increased °Brix values, ranging from 4.60 to 4.75 ± 0.15, compared to all other treatments and control (*p* < 0.05). PME and PG activity was estimated as 15.17 and 19.01 units/mL for control samples, respectively. All PGPB treatments resulted in increased PME activity, ranging from 19.58 to 24.94 units/mL (*p* < 0.05) compared to that of control samples. Application of *B. mojavensis, B. velezensis, and Mix* resulted in higher PG activities of the final tomatoes by 58.0, 34.5, and 71.2% compared to untreated samples. *B. licheniformis*, *B. pumilus*, and *B. subtilis* application resulted in 10–25% PG activity decrease that is important for the tomato industries. *B. licheniformis, P. megaterium*, *B. subtilis, and Azotobacter* treatments resulted in statistically significant increased total carotenoids content by 22.8, 25.1, 26.4, and 26.8%, respectively, compared to that of control samples (5.82 ± 0.35 mg/g dw). From PGPB treatments, the highest lycopene content was observed for the *P. megaterium* treated tomatoes (20.64 ± 3.35 μg/g dw), followed by *B. licheniformis, B. subtilis*, and *B. amyloliquefaciens* treated tomatoes (18.12, 18.75 and 19.05 μg lycopene/g dw, respectively). Not significant effect was observed for total phenolic content of samples (values ranged from 4.12 to 4.85 mg CAE/g dw for all samples). *B. licheniformis, B. subtilis*, and *B. amyloliquefaciens* treated tomatoes possessed an increased antioxidant activity (values ranged from 3.71 to 3.78 mg Trolox/g dw) with statistically significant differences compared to that of control samples (2.88 ± 0.07 mg Trolox/g dw) and all other PGPB treated samples.

## 4. Discussion

The results obtained showed that the soil application of PGPB on industrial tomato enhanced the plant growth and plant physiology, increased the yield, and improved the quality characteristics of the industrial tomato.

At the final measurement of dry weight per plant, *B. licheniformis* and *B. subtilis* treatments led to an increase equal to 39.38% and 32.23%, respectively, compared to that of control. Concerning photosynthetic rate, the use of *P. megaterium* increased the rate from 5.54% to 25.73%, compared to that of the control, at the third and the first measurement respectively. At the final measurement, *B. velezensis*, and *A. chroococcum* gave values higher than that of the control. For the transpiration rate, at the second measurement, most PGPB treatments resulted in higher values compared to that of control, except *B. amyloliquefaciens*, *A. chroococcum*, and *P. megaterium*. In a similar research, Masood et al., [38] performed two pot experiments to assess the effect of *Bacillus pumilus* to the growth of tomato plants when it is combined with N fertilization. In their results they found that when this PGPB was inoculated, it improved significantly in shoot dry weight, leaf transpiration, and photosynthesis, especially when the additional N fertilization was applied. *Bacillus pumilus* was also put on a test for Boron (B) toxicity [39], and researchers found that this PGPB increased tomato plant’s antioxidation activity that helped them tolerate B toxicity and enhancing their growth under these stress conditions. Lee et al. [40] used at tomato plants and three other vegetable crops the PGPR strain BS21-1 of *Bacillus subtilis* to investigate the effect of this strain in plant growth and diseases suppression. In particular, the use of strain BS21-1 resulted in a significant increase at the tomato’s plants height as well as leaf width in both organic soil and seed bed soil conditions. In a similar experiment García et al. [41] tested a strain of *Bacillus licheniformis* on two vegetable crops to assess their growth and disease suppression when this strain was inoculated. In the two varieties of tomato plants that were tested (*Daniela* and *Brillante)*, stem and leaves dry weight, plant height, and leaf area presented an increase to their values compared to that of the control, but were not statistically significant. However, a significant increase was found in the number and the diameter of fruits compared to that of the control 45 days after the last inoculation. Another strain of PGPR was used by Mayak et al. [42] to explore the effect at the resistance of tomato plants when they are cultivated under salt stress. The strain *Achromobacter piechaudii* ARV8 performed well under water stress among the other 6 PGPR strains that were tested. It resulted not only in statistically significant differences in shoot and root length and fresh and dry weight of seedlings, but also in the Water Use Efficiency which is the ratio of the total biomass to the transpiration.

At the measurement of mean fruit weight per plant, *B. subtilis*, *B. amyloliquefaciens, P. megaterium*, and *B. licheniformis* resulted in values increased by 30.70%, 28.81%, 27.52%, and 26.78% compared to that of control, respectively. As for the yield per plant, the use of *B. licheniformis* and *B. subtilis* increased by 51.94% and 45.87%, respectively. The use of PGPB increased by 10–19% the °Brix compared to that of control. The value of total soluble solids (°Brix) of tomatoes is usually dependent to tomato variety and stage of maturity. Decreased values of Brix indicates loss of tomato quality [43], especially for industrial tomatoes that are used for the production of tomato products (paste, concentrated juice, etc.) and total solids need to be as high as possible for higher yields. Concerning PME activity, PGPB treatments resulted in increased activity compared to that of control, while *B. mojavensis*, *B. velezensis*, and *Mix* resulted in higher PG activities of the final tomatoes up to 71.2%. PME and PG are two endogenous enzymes in tomato related to texture and consistency of juices. In tomato industries, it is necessary to control the activity of those two pectinolytic enzymes for higher quality concentrated tomato products. Synergistic action of PME and PG leads to degradation of juice viscosity and to serum loss. The obtained results showed that the application of certain bio-stimulants may increase the bioactive compounds of harvested tomatoes, such as total carotenoids, especially lycopene and total phenolic compounds. Lycopene is the major carotenoid compound (80–86% of total carotenoids in tomatoes) responsible for the red color to tomato fruit [44], and for the antioxidant activity of tomatoes [45]. Lycopene content of control harvested tomatoes was 13.75 ± 3.35 μg/g d.m, which is in agreement with previous works [46]. The antioxidant activity of PGPB treated tomatoes was well correlated to the total carotenoid compounds and especially lycopene content. The increased antioxidant activity might be attributed due to the higher carotenoids, lycopene, and phenolic content [34] of *B. licheniformis, B. subtilis*, and *B. amyloliquefaciens* treated tomatoes.

The effect of PGPB on the yield and quality characteristics of tomato is a subject of rising interest for the research community, as it could contribute for a more sustainable agriculture. In similar research on industrial tomato plants, Bona et al. [20] tested *Pseudomonas* sp. 19Fv1T and *P. fluorescens* C7 strains while applying the 70% of the common practice fertilization, and they found that both strains presented great results for fruit fresh weigh and at sweetness index when the *Pseudomonas* sp. 19Fv1T strain was used; these parameters were even better than that of the control. Even though 70% of fertilization was used when the PGPB strains were used, the lycopene as well as the β-carotene were not statistically significantly different. Taking a step further from the previous experiment, Bona et al. [47] combined the same PGPB strains with Arbuscular Mycorrhizal Fungi (AMF) and found that the combination of those organisms while having 70% of the traditional fertilization presented promising results under those conditions. The number of fruits and the total weight were increased compared to that of the control with 70% of fertilization except when *Pseudomonas* sp. 19Fv1T strain combined alone with AMF. Also, they presented better results than that of the control for lycopene and the β-carotene measurements. *Kosakonia radicincitans* were inoculated on tomato seeds by Berger et al. [19] and were found to increase significantly the mass of tomato fruit (+24%) and the fruit per plant (+18%), however, they presented decreased levels of lycopene, lutein, and β-carotene. Dudás et al. [18] evaluated the use of 3 products that contain strains of PGPB. The product that contained the strain *Bacillus amyloliquefaciens* FZB42 presented significant better results in the pot experiment in case of total soluble solid (TSS) content of tomato fruits compared to that of the control and to the other products that were used while in the field environment the TSS were significant increased compared to that of the control but not significant different from the other PGPB products. In a greenhouse experiment Lee et al. [21] evaluated the *Rhodopseudomonas* sp. strains BL6 and KL9 on tomato plant growth and quality characteristics. They found that *Rhodopseudomonas* sp. KL9 strain had an increased average fresh weight of fruit than the control and also a higher lycopene content. However, even though *Rhodopseudomonas* sp. BL6 strain had also higher fruit weight, the lycopene content was lower than that of the control.

**Figure 4:**
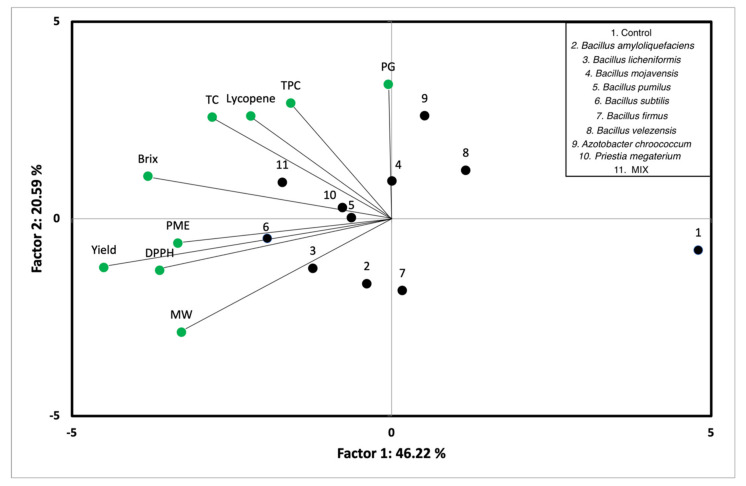
Biplot of principal component analysis of application of different PGPB strains on cultivation of industrial tomato. PG: poligalacturonase activity; TPC: total phenolic compounds; TC: total carotenoids; PME: pectin methylesterase activity; DPPH: sntioxidant activity; MW: mean fruit weight. Mix: mix of *P. megaterium* + *Azotobacter chroococcum*.

To investigate the correlation of the yield, mean fruit weight and quality characteristics by using different PGPB on the cultivation of industrial tomato, principal components analysis (PCA) was used (Figure 4). Each point on the loading plot represented the contribution of a variable (yield, mean fruit weight, and quality characteristics: total soluble solids (°Brix), total carotenoids, lycopene concentration, total phenolic compounds, antioxidant activity, PME, and PG activity) to the score, while each point on the score plot represented a tested sample. The first principal component (PC1) describes 46.22% of the variation of experiments, whereas the second principal component (PC2) decribes 20.59%, respectively, so that they contributed 66.81% of total variation of experiments.

According to the PCA plot, the total soluble solids (°Brix), antioxidant activity, PME activity and yield had negative effect on PC1, while the lycopene concentration, total phenolic compounds, and PG activity had positive effect on PC2. Furthermore, there are correlations between the antioxidant activity, PME activity, and between the lycopene concentration, total phenolic compounds and total carotenoids. Based on PCA score plot of the tested samples, 5 main groups of samples were noted. The groups are (a) *B. mojavensis, A. chroococcum, B. velezensis* (b) *Mix, P. megaterium, B. pumilus* (c) *B. subtilis*, *B. licheniformis* (d) *B. amyloliquefaciens*, *B. pseudomycoides*, and (e) control.

The samples of group (a) confirmed that *B. mojavensis*, *A. chroococcum*, and *B. velezensis* were the most effective treatments for the cultivation of industrial tomato, giving tomatoes with the highest PG activity. The samples of group (b) indicated that *Mix, P. megaterium* and *B. pumilus* treatments resulted in harvested tomatoes with high lycopene, total phenolic compounds, and total carotenoids concentration as well as total soluble solids (°Brix). Many studies demonstrated that the treatment of industrial tomato plants with PGPR including *P. megaterium* affected positively the quality characteristics of the tomato products in terms of lycopene and phenolic content as well as Brix degree [48,49]. The samples of group (c) showed that *B. subtilis* and *B. licheniformis* treatments resulted in tomatoes with high yield and the maximum PME and antioxidant activity compared with that of the other treatments. This finding is in accordance with other studies indicating that *B. licheniformis* treatment increased the antioxidant activity of greenhouse cultivated tomato fruits (*Solanum lycopersicum* L. var. Sheva) [50] and *B. subtilis* CBR05 treatment enhanced the yield and the quality of tomato fruits produced under greenhouse conditions [51]. Moreover, the samples of group (d) showed that tomatoes harvested by applying *B. amyloliquefaciens* and *B. pseudomycoides* treatments presented similar characteristics in terms of lycopene, total phenolic compounds, and total carotenoids concentration as well as total soluble solids (°Brix). The sample of group (e) indicated that the untreated tomato plants (control) indicated that the harvested tomatoes showed the lowest yield, mean fruit weight, and the lowest quality characteristics concentration in terms of total soluble solids (°Brix), total carotenoids, lycopene, total phenolic compounds, antioxidant activity, PME, and PG activity. Concluding, the results depicted by principal components analysis are in agreement with those discussed above.

## 5. Conclusions

The plant growth promoting bacteria (PGPB) strains that were applied via soil application as biostimulants in this study presented a significant effect not only on growth and yield but also on the physiology and the quality characteristics of tomato plants. The dry weight of the tomato plants was positively affected by most of the bacteria strains that were used, with the *B. licheniformis* standing above them. Photosynthetic rate was also affected positively by the application of bacterial strains, and in particular, *P. megaterium* presented greater results. *B. subtilis*, *B. amyloliquefaciens, P. megaterium*, and *B. licheniformis* resulted in values increased by 30.70%, 28.81%, 27.52%, and 26.78% higher than that of the control, respectively, regarding the mean fruit weight per plant. As for the yield per plant, the use of *B. licheniformis* and *B. subtilis* increased by 51.94% and 45.87%, respectively, compared to that of the control. As for the quality characteristics, interestingly, there was an improvement in the quality of the fruits in nearly every bacteria strain. In particular, *B. pumilus and the* mix of *P. megaterium* and *A. chroococcum* had the most increased total soluble solids in the tomato fruits, while *P. megaterium* had the best content in lycopene and total carotenoids, *B. pseudomycoides* was the bacteria with the most increased PME activity and *B. mojavensis* along with the mix of *P. megaterium*, and *A. chroococcum* the greatest increases in PG activity. What is really interesting is that nearly all the bacteria strains had an increased antioxidant activity significantly better that that of the control. This study proved that the use of PGPB as biostimulants can improve plant growth, physiology of the plant, yield, and quality characteristics of the industrial tomato. More studies should be performed in various crops and on various soil and climatic conditions to understand more clearly how the use of PGPB can be implemented as an agricultural practice for the increase of yield and quality. The adaptability of such microbial inoculants in open field experiments under variable soil and climatic conditions will be a major challenge for the researchers in future studies.

## Figures and Tables

**Table 1:** Description of strain, NCBI accession number, pH, colony forming unit (CFU), and auxin concentration of PGPB used.

Identification	Strain	NCBI Accession Number	pH	CFU/mL	Concentration of Auxin (ppm)
*Bacillus amyloliquefaciens*	B002	MW562326	6.70	6.5 × 10^9^	38.45
*Bacillus licheniformis*	B017	MW562833	6.15	6.0 × 10^9^	45.00
*Bacillus mojavensis*	B010	MW562828	5.95	4.1 × 10^9^	40.52
*Bacillus pumilus*	W27-4	MW562832	6.01	2.6 × 10^9^	58.10
*Bacillus subtilis*	Z3	MW396734	5.99	3.0 × 10^9^	43.97
*Bacillus pseudomycoides*	S3	MW687620	5.92	6.0 × 10^9^	39.14
*Bacillus velezensis*	B006	MW562831	6.08	5.2 × 10^8^	46.03
*Azotobacter chroococcum*	A004	-	7.20	6.4 × 10^9^	24.00
*Priestia megaterium*	B004	MW562819	6.40	6.2 × 10^9^	57.76
Mix					

The sequence data of the strains with accession number were submitted to GenBank of NCBI. Mix: mix of *Priestia megaterium* B004 (3.4 × 10^7^ CFU/mL) + *Azotobacter chroococcum* A004 (1.3 × 10^7^ CFU/mL) with zeolite as a carrier.

**Table 2:** Physical and chemical properties of soil.

Parameters	Values
Sand (%)	34
Silt (%)	28
Clay (%)	38
Soil Texture	Clay Loam
pH	7.6
Saturation percentage (%)	55
Electrical Conductivity (mS cm^−1^)	1.41
Total salts (%)	0.05
Organic Matter (%)	4.9
Total Nitrogen (mg g^−1^)	2.2
Available K (cmoℓ+ kg^−1^)	1.2
Available Ca (cmoℓ+ kg^−1^)	22
Available Mg (cmoℓ+ kg^−1^)	6.4
Available P (mg kg^−1^)	87
Fe-DTPA (mg kg^−1^)	34
Cu-DTPA (mg kg^−1^)	3.7
Zn-DTPA (mg kg^−1^)	8.2
Mn-DTPA (mg kg^−1^)	15.7
Available B (mg kg^−1^)	1.5

**Table 3:** Effect of PGPB strains on dry weight of whole plants 66, 80, and 94 DAT.

Treatment	Dry Weight (g per Plant)		
	66 DAT	80 DAT	94 DAT
Control	243 ± 5 ^de^	361 ± 16 ^d^	546v24 ^d^
*B. amyloliquefaciens*	289 ± 6 ^bc^	403 ± 21 ^bc^	659 ± 65 ^bc^
*B. licheniformis*	328 ± 10 ^a^	442 ± 19 ^a^	761 ± 11 ^a^
*B. mojavensis*	301 ± 21 ^b^	402 ± 24 ^bc^	624 ± 18 ^cd^
*B. pumilus*	305 ± 18 ^b^	389 ± 6 ^cd^	697 ± 45 ^abc^
*B. subtilis*	304 ± 13 ^b^	381 ± 24 ^cd^	722 ± 68 ^ab^
*B. pseudomycoides*	336 ± 23 ^a^	388 ± 24 ^cd^	683 ± 24 ^abc^
*B. velezensis*	268 ± 5 ^cd^	371 ± 21 ^cd^	627 ± 15 ^cd^
*A. chroococcum*	256 ± 10 ^de^	361 ± 16 ^d^	699 ± 88 ^abc^
*P. megaterium*	275 ± 10 ^cd^	379 ± 20 ^cd^	695 ± 43 ^abc^
Mix	334 ± 13 ^a^	436 ± 19 ^ab^	702 ± 45 ^abc^
F_treat_	16.513 ^***^	5.597 ^***^	4.572 ^***^

DAT: Days After Transplanting. Mix: Mix of *Priestia megaterium* B004 + *Azotobacter chroococcum* A004 with zeolite as a carrier. Means followed by the same letter for treatments are not significantly different according to Duncan test (*p* < 0.05). Values presented are mean values of three replicates ± standard deviation. Significance levels: *** *p* < 0.001.

**Table 4:** Effect of PGPB strains on photosynthetic rate 66, 80, and 94 DAT.

Treatment	Photosynthetic Rate (μmol CO_2_ m^−2^ s^−1^)		
	66 DAT	80 DAT	94 DAT
Control	14.34 ± 0.20 ^d^	15.38 ± 0.25 ^e^	16.62 ± 0.23 ^de^
*B. amyloliquefaciens*	17.19 ± 0.41 ^b^	18.00 ± 0.19 ^ab^	17.38 ± 0.58 ^abcd^
*B. licheniformis*	17.41 ± 0.51 ^ab^	17.09 ± 0.48 ^cd^	17.01 ± 0.32 ^cde^
*B. mojavensis*	16.07 ± 0.44 ^c^	16.72 ± 0.47 ^d^	16.38 ± 0.45 ^e^
*B. pumilus*	16.75 ± 0.47 ^bc^	15.87 ± 0.28 ^e^	17.32 ± 0.40 ^abcde^
*B. subtilis*	17.96 ± 0.44 ^a^	17.75 ± 0.19 ^b^	16.94 ± 0.61 ^cde^
*B. pseudomycoides*	17.29 ± 0.39 ^ab^	17.43 ± 0.08 ^bc^	17.22 ± 0.81 ^bcde^
*B. velezensis*	16.13 ± 0.27 ^c^	16.82 ± 0.34 ^cd^	18.02 ± 0.35 ^ab^
*A. chroococcum*	16.93 ± 0.47 ^b^	16.79 ± 0.54 ^cd^	17.82 ± 0.64 ^abc^
*P. megaterium*	18.03 ± 0.38 ^a^	18.48 ± 0.15 ^a^	18.27 ± 0.41 ^a^
Mix	17.38 ± 0.36 ^ab^	17.81 ± 0.60 ^b^	17.54 ± 0.64 ^abcd^
F_treat_	20.161 ^***^	19.264 ^***^	3.680 ^***^

DAT: Days After Transplanting. Mix: Mix of *Priestia megaterium* B004 + *Azotobacter chroococcum* A004 with zeolite as a carrier. Means followed by the same letter for treatments are not significantly different according to Duncan test (*p* < 0.05). Values presented are mean values of three replicates ± standard deviation. Significance levels: *** *p* < 0.001.

**Table 5:** Effect of PGPB strains on transpiration rate 66, 80, and 94 DAT.

Treatment	Transpiration Rate (mmol H_2_O m^−2^ s^−1^)		
	66 DAT	80 DAT	94 DAT
Control	2.46 ± 0.08 ^f^	2.51 ± 0.22 ^c^	3.23 ± 0.05
*B. amyloliquefaciens*	3.38 ± 0.21 ^abc^	2.75 ± 0.09 ^bc^	3.44 ± 0.15
*B. licheniformis*	3.29 ± 0.21 ^bc^	2.98 ± 0.20 ^ab^	3.68 ± 0.41
*B. mojavensis*	3.35 ± 0.12 ^bc^	3.09 ± 0.18 ^ab^	3.60 ± 0.29
*B. pumilus*	2.93 ± 0.27 ^de^	3.23 ± 0.42 ^a^	3.52 ± 0.37
*B. subtilis*	3.71 ± 0.13 ^a^	3.05 ± 0.20 ^ab^	3.39 ± 0.25
*B. pseudomycoides*	3.04 ± 0.31 ^cde^	2.94 ± 0.24 ^ab^	3.78 ± 0.12
*B. velezensis*	3.23 ± 0.05 ^bcd^	3.25 ± 04 ^a^	3.59 ± 0.12
*A. chroococcum*	2.77 ± 0.12 ^ef^	2.74 ± 0.10 ^bc^	3.64 ± 0.21
*P. megaterium*	3.55 ± 0.26 ^ab^	2.88 ± 0.05 ^abc^	3.45 ± 0.38
Mix	3.40 ± 0.10 ^ab^	3.02 ± 0.25 ^ab^	3.36 ± 0.23
F_treat_	11.119 ^***^	3.404 ^***^	1.152 ^ns^

DAT: Days After Transplanting. Mix: Mix of *Priestia megaterium* B004 + *Azotobacter chroococcum* A004 with zeolite as a carrier. Means followed by the same letter for treatments are not significantly different according to Duncan test (*p* < 0.05). Values presented are mean values of three replicates ± standard deviation. Significance levels: ***: *p* < 0.001; ns: not significant (*p* > 0.05).

**Table 6:** Effect of PGPB strains on total soluble solids (°Brix), PME and PG activity (units/mL), total carotenoids (mg /g d.m), total phenolic compounds (mg of CAE/g d.m), lycopene concentration (μg /g d.m) and antioxidant activity (mg Trolox/g d.m) of harvested tomato.

Samples	°Brix	PME Activity (units/mL)	PG Activity (units/mL)	Total Carotenoids (mg /g d.m)	Total Phenolic Compounds (mg of CAE/g d.m)	Lycopene (μg/g d.m)	Antioxidant Activity (mg Trolox/g d.m)
*Control*	4.00 ± 0.05 ^d^	15.17 ± 1.95 ^d^	19.01 ± 1.60 ^c^	5.82 ± 0.35 ^c^	4.12 ± 0.01	13.75 ± 3.35 ^cd^	2.88 ± 0.07 ^bc^
*B. amyloliquefaciens*	4.60 ± 0.00 ^ab^	19.58 ± 2.50 ^bc^	16.28 ± 0.81 ^cd^	7.15 ± 0.86 ^ab^	4.23 ± 0.18	19.05 ± 2.52 ^ab^	3.78 ± 0.64 ^a^
*B. licheniformis*	4.70 ± 0.13 ^a^	19.66 ± 2.74 ^bc^	14.46 ± 4.05 ^d^	6.42 ± 0.91 ^abc^	4.35 ± 0.41	18.12 ± 3.12 ^ab^	3.71 ± 0.35 ^a^
*B. mojavensis*	4.60 ± 0.15 ^ab^	22.83 ± 1.63 ^ab^	30.09 ± 1.40 ^a^	6.76 ± 0.77 ^abc^	4.43 ± 0.32	17.19 ± 2.82 ^abc^	3.45 ± 0.36 ^ab^
*B. pumilus*	4.75 ± 0.05 ^a^	20.52 ± 2.61 ^bc^	16.92 ± 4.31 ^cd^	6.27 ± 0.45 ^abc^	4.53 ± 0.10	15.32 ± 4.35 ^acd^	3.22 ± 0.25 ^abc^
*B. subtilis*	4.50 ± 0.10 ^bc^	22.34 ± 3.40 ^abc^	16.35 ± 0.52 ^cd^	7.36 ± 0.28 ^a^	4.85 ± 0.10	18.75 ± 0.10 ^ab^	3.77 ± 0.32 ^a^
*B. pseudomycoides*	4.50 ± 0.04 ^bc^	24.94 ± 3.27 ^a^	24.80 ± 1.99 ^b^	6.13 ± 0.52 ^bc^	4.12 ± 0.31	11.34 ± 3.54 ^d^	3.23 ± 0.43 ^abc^
*B. velezensis*	4.40 ± 0.02 ^c^	19.64 ± 1.34 ^cd^	28.97 ± 3.01 ^ab^	7.04 ± 0.44 ^ab^	4.48 ± 0.36	16.42 ± 4.25 ^abc^	3.37 ± 0.16 ^a^
*A. chroococcum*	4.60 ± 0.07 ^ab^	18.24 ± 2.08 ^ad^	25.04 ± 1.96 ^b^	7.38 ± 0.95 ^a^	4.73 ± 0.26	16.84 ± 2.19 ^abc^	2.62 ± 0.35 ^c^
*Mix*	4.70 ± 0.13 ^a^	21.23 ± 0.94 ^abc^	32.53 ± 0.79 ^a^	7.21 ± 0.29 ^ab^	4.48 ± 0.20	18.70 ± 4.35 ^ab^	3.56 ± 0.57 ^ab^
*P. megaterium*	4.60 ± 0.03 ^ab^	20.53 ± 1.06 ^bc^	24.84 ± 1.62 ^b^	7.28 ± 0.44 ^a^	4.19 ± 0.27	20.64 ± 3.81 ^a^	3.45 ± 0.27 ^ab^
F_treat_	19.560 ***	3.841 **	21.179 ***	3.384 **	2.496 ^ns^	3.650 **	3.824 **

*Mix*: Mix of *P. megaterium* + *Azotobacter chroococcum*. Different superscript letters indicate significantly different means (*p* < 0.05) within a column according to Duncan test. Significance levels: ***: *p* < 0.001; **: *p* < 0.01; ns: not significant (*p* > 0.05).

## Data Availability

All data generated or analyzed during this study are included in this published article. Further inquiries can be addressed to the corresponding author.
